# Inspiration4 data access through the NASA Open Science Data Repository

**DOI:** 10.1038/s41526-024-00393-5

**Published:** 2024-05-14

**Authors:** Lauren M. Sanders, Kirill A. Grigorev, Ryan T. Scott, Amanda M. Saravia-Butler, San-huei Lai Polo, Rachel Gilbert, Eliah G. Overbey, JangKeun Kim, Christopher E. Mason, Sylvain V. Costes

**Affiliations:** 1 Space Biosciences Research Branch, NASA Ames Research Center, Moffett Field, CA USA; 2 Blue Marble Space, Seattle, WA USA; 3 KBR, Houston, TX USA; 4 Department of Physiology and Biophysics, Weill Cornell Medicine, New York, NY USA; 5 The HRH Prince Alwaleed Bin Talal Bin Abdulaziz Alsaud Institute for Computational Biomedicine, Weill Cornell Medicine, New York, NY USA; 6 Center for STEM, University of Austin, Austin, TX USA; 7 The WorldQuant Initiative for Quantitative Prediction, Weill Cornell Medicine, New York, NY USA

**Keywords:** Computational biology and bioinformatics, Biological techniques

## Abstract

The increasing accessibility of commercial and private space travel necessitates a profound understanding of its impact on human health. The NASA Open Science Data Repository (OSDR) provides transparent and FAIR access to biological studies, notably the SpaceX Inspiration4 (I4) mission, which amassed extensive data from civilian astronauts. This dataset encompasses omics and clinical assays, facilitating comprehensive research on space-induced biological responses. These data allow for multi-modal, longitudinal assessments, bridging the gap between human and model organism studies. Crucially, community-driven data standards established by NASA’s OSDR Analysis Working Groups empower artificial intelligence and machine learning to glean invaluable insights, guiding future mission planning and health risk mitigation. This article presents a concise guide to access and analyze I4 data in OSDR, including programmatic access through GLOpenAPI. This pioneering effort establishes a precedent for post-mission health monitoring programs within space agencies, propelling research in the burgeoning field of commercial space travel’s impact on human physiology.

## Introduction

The increased accessibility of commercial and private spaceflight travel has made it imperative to understand the short- and long-term effects of space stressors on human health. The NASA Open Science Data Repository (OSDR)^1–3^ provides open and FAIR^4^ access to spaceflight and space-relevant biological studies from the past decades. Research enabled by OSDR data has revealed a complex network of molecular and physiological effects of spaceflight across living systems, from microbes to plants to mammals (https://osdr.nasa.gov/bio/data/publications.html). Most prior research using OSDR data has focused on model organisms such as rodents, worms, and fruit flies. These valuable measurements provide insight into the response of neuromuscular, immune, and developmental biosystems to the stressors and hazards of spaceflight. Integrating human in vivo data takes these studies one step further and map previously characterized model organisms’ biological responses to the limited knowledge on human physiology in space.

The 2021 SpaceX Inspiration4 (I4) mission collected a comprehensive atlas of biological measurements from four civilian astronauts, providing a wealth of data to characterize the effects of spaceflight on the human body. In the current special *Nature* package titled “The Second Space Age: Omics, Platforms, and Medicine Across Orbits,” a wide battery of analyses of these data have already provided valuable insights (Mason et al., *Nature*. 2023).

The I4 mission stands as a significant milestone, amassing an extensive collection of biological measurements from four civilian astronauts, thereby adding an invaluable series of datasets for deciphering the repercussions of spaceflight on human health. The open access of these datasets in the NASA OSDR provides a unique opportunity for the scientific community, citizen scientists, researchers, and students to continue using OSDR resources to further unlock profound insights into the consequences of space travel on the human body. In this short article, we guide the readers on how to best conduct future analysis by presenting a series of vignettes illustrating pathways for accessing metadata and data from this rich resource through OSDR. We hope these guidelines will help the community contribute further to the vast compendium of findings already published from OSDR data and enable new hypotheses to be tested with both the human and model organism data.

Compared to traditional model organisms used for research, one unique aspect of these data is the breadth of longitudinal measurements available from several human astronauts. Such temporal information for several individuals is unprecedented and pushes further the well-known “NASA’s Twin Study”^5^ where a single astronaut was monitored over 340 days on the international space station, referred to as a “N of 1” experiment. In contrast, even though the duration of the I4 mission was much shorter (3 days), it was at a higher altitude (590 km vs. 420 km), and since it featured four individuals monitored with such biological depth, the data opens the door to new and innovative follow-up studies (e.g. Polaris Dawn missions).

Incorporating environmental data with microbiome data and a wide variety of phenotypic and high-throughput sequencing assays provides a rare opportunity for multi-modal, longitudinal assessment of short-term and long-term spaceflight effects on humans. These datasets also provide a much-needed ability to compare human spaceflight effects to the previously characterized spaceflight consequences in model organisms.

The analyses conducted thus far on the I4 mission’s data have already yielded substantial revelations, shedding light on the intricate interplay between space stressors and the immune system, cell-specific responses to spaceflight, rapid microbial interchange of the crew, and detailed maps of the changes in the skin from astronauts (Overbey et al., Nature 2023; Tierney et al., Nature Microbiology 2023; Kim et al., Nature 2023). These analyses demonstrate the great potential of these data for future knowledge. The inclusion of multi-modal data, such as the blood datasets, which cover a diverse array of measurements (OSD-569, −570, −571, −575), also presents an exciting avenue for future exploration. This broader scope for analysis could unveil deeper connections between different biological responses and offer a more comprehensive understanding of the body’s adaptations to the space environment. Combining omics and medical assay data is the first step to understanding the biological significance of integrated findings. Translating them into actionable insights for space health will be our next step, which remains challenging but, for the first time, attainable.

Looking ahead, integrating artificial intelligence and machine learning (AI/ML) in the analysis of biomedical data from the I4 mission also holds great promise. With all OSDR data accessible in a cloud-based public repository, standardized metadata, and a robust API query system, this effort demonstrates AI readiness and empowers our user community to explore beyond current findings. The sheer volume and complexity of the data call for advanced computational approaches that can uncover patterns, correlations, and anomalies that might otherwise remain hidden. Powerful techniques, such as machine learning and deep learning, hold the potential to extract meaningful but complex insights from the vast dataset, facilitating the identification of subtle trends and potential health risks associated with space travel. NASA OSDR’s commitment to FAIR practices ensures that researchers across the globe can harness the power of AI to conduct cutting-edge analyses, fostering collaboration and accelerating the pace of discovery. Finally, this work sets a precedent for space agencies to establish comprehensive, post-mission health monitoring programs, incorporating omics and medical assay data, while informing future mission planning and health risk mitigation strategies.

## Methods

**Fig. 1:**
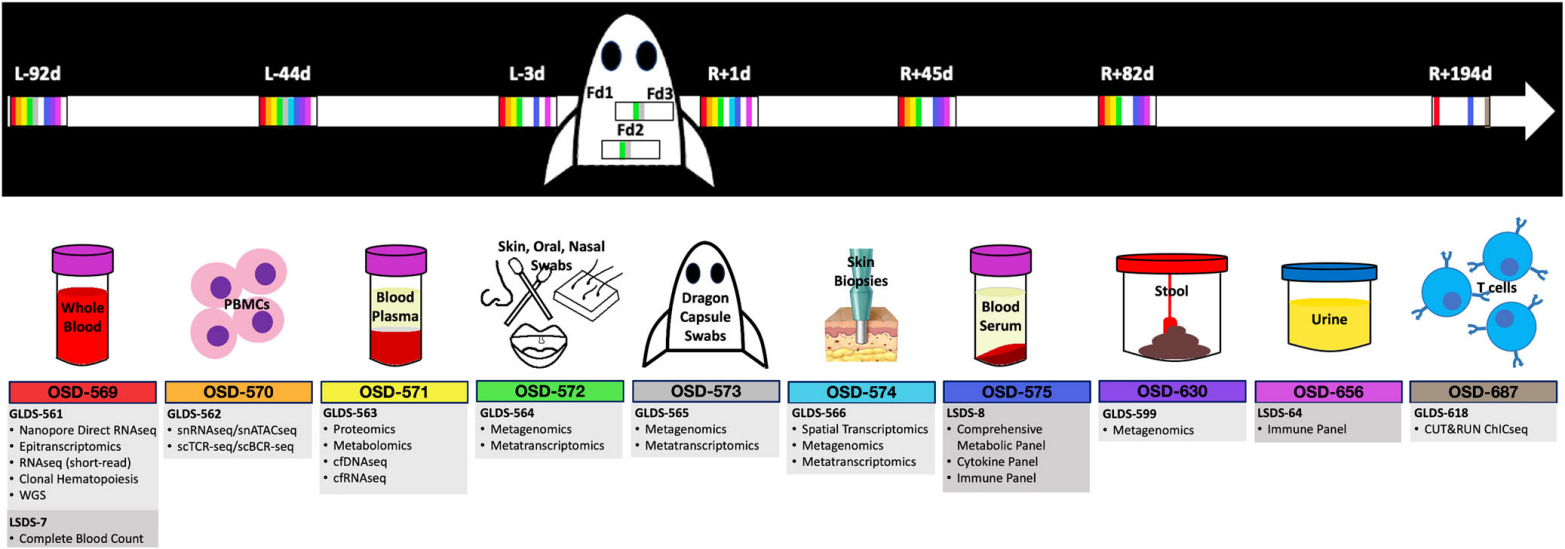
Overview of available I4 data in OSDR across 10 studies separated by tissue. I4 mission data are separated into 10 OSD studies based on sample/tissue type as shown above. For each OSD, the associated omics and non-omics data are listed under the GLDS-ID (light gray) and LSDS-ID (dark gray), respectively. The time points samples were collected for each OSD are color-coded in the I4 mission timeline at the top. L Launch, F Flight, R Return, d Days.

In the realm of scientific inquiry involving I4 data, it is imperative to note that publicly accessible information pertains solely to processed data, given that raw data pertaining to individual human subjects, encompassing genetic sequence data among other facets, necessitate an application procedure that mandates approval from an ethical oversight board for access. The protocol for controlled data access has been meticulously devised, drawing inspiration from established guidelines within the Database of Genotypes and Phenotypes (dbGAP) and incorporating best practices derived from the United Kingdom Biobank. This comprehensive framework encompasses an expansive array of data and sample categories, as illustrated in Fig. 1.

These data are captured across eight studies, delineated by tissue and sample type, and includes omics assays such as direct RNA sequencing (RNA-seq), single nuclei ATAC-seq and RNA-seq, metagenomics/metatranscriptomics, spatial transcriptomics, and proteomics to comprehensive metabolic and immune panels. Moreover, clinical assays for blood profiling, such as complete blood count (CBC) and metabolic panels, are also included in the data resource.

All subjects were consented at an informed consent briefing (ICB) at SpaceX (Hawthorne, CA), and samples were collected and processed under the approval of the Institutional Review Board (IRB) at Weill Cornell Medicine, under Protocol 21-05023569. All crew members have consented for data and sample sharing.

### Reporting summary

Further information on research design is available in the Nature Research Reporting Summary linked to this article.

## Data records

For illustration purposes, a single dataset will be used as an example for researcher access of I4 data from OSDR: OSD-572, which holds metagenomics and metatranscriptomics data from crew skin samples collected to assess the response of microbial populations (skin, oral, nasal) to spaceflight. Samples were collected at three timepoints pre-flight (launch minus 92 days (L-92), L-44, and L-3), mid-flight for three time points (flight day 1 (FD1), FD2, FD3), and post-flight (return plus 1 day (R + 1), R + 45 R + 82).

The OSDR study page (https://osdr.nasa.gov/bio/repo/data/studies/OSD-572) contains several sections with information about the study, including a description of the experiment that gave rise to the data, associated experiments, payloads, and missions, and a section detailing each experimental or analytical protocol involved in generating the dataset. A “publications” section also lists all published papers associated with this study.

**Fig. 2:**
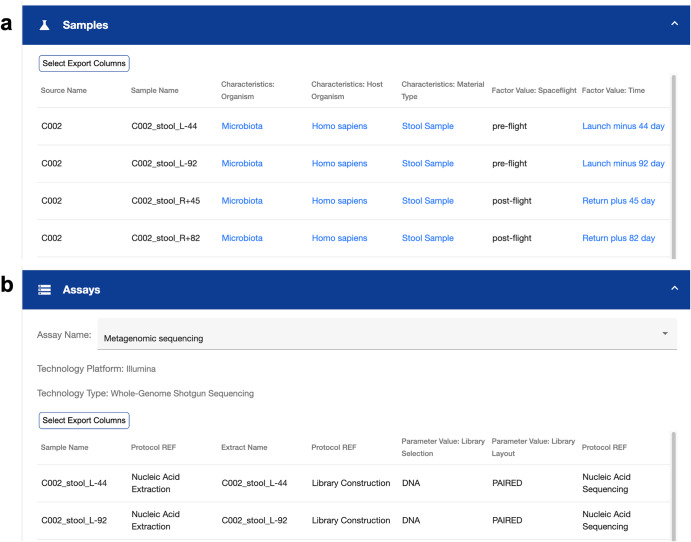
Sample and assay metadata available in OSDR for I4 study OSD-572. **a** Sample metadata table contains sample level information regarding sample characteristics and experimental factors. **b** Assay metadata table contains information about each assay on a per sample level.

Figure 2 shows snapshots of the repository, emphasizing the metadata tables available for OSD-572. The *Samples* table (Fig. 2a) contains metadata information on each sample, including intrinsic characteristics of the sample (e.g., host organism, material type) as well as factor values relating to the experiment (e.g., spaceflight status, time collected). The *Assays* table contains metadata information for each sample pertaining to the assay specified in the drop-down menu (Fig. 2b). Users can choose a specific assay to display from the drop-down menu, and the assay table will update accordingly.

Both metadata tables can be downloaded as a comma-separated-values (CSV) file with user-selected columns to aid downstream analyses. Sample names map across metadata and data fields to enable targeted selection of specific samples or differential analysis across groups.

**Fig. 3:**
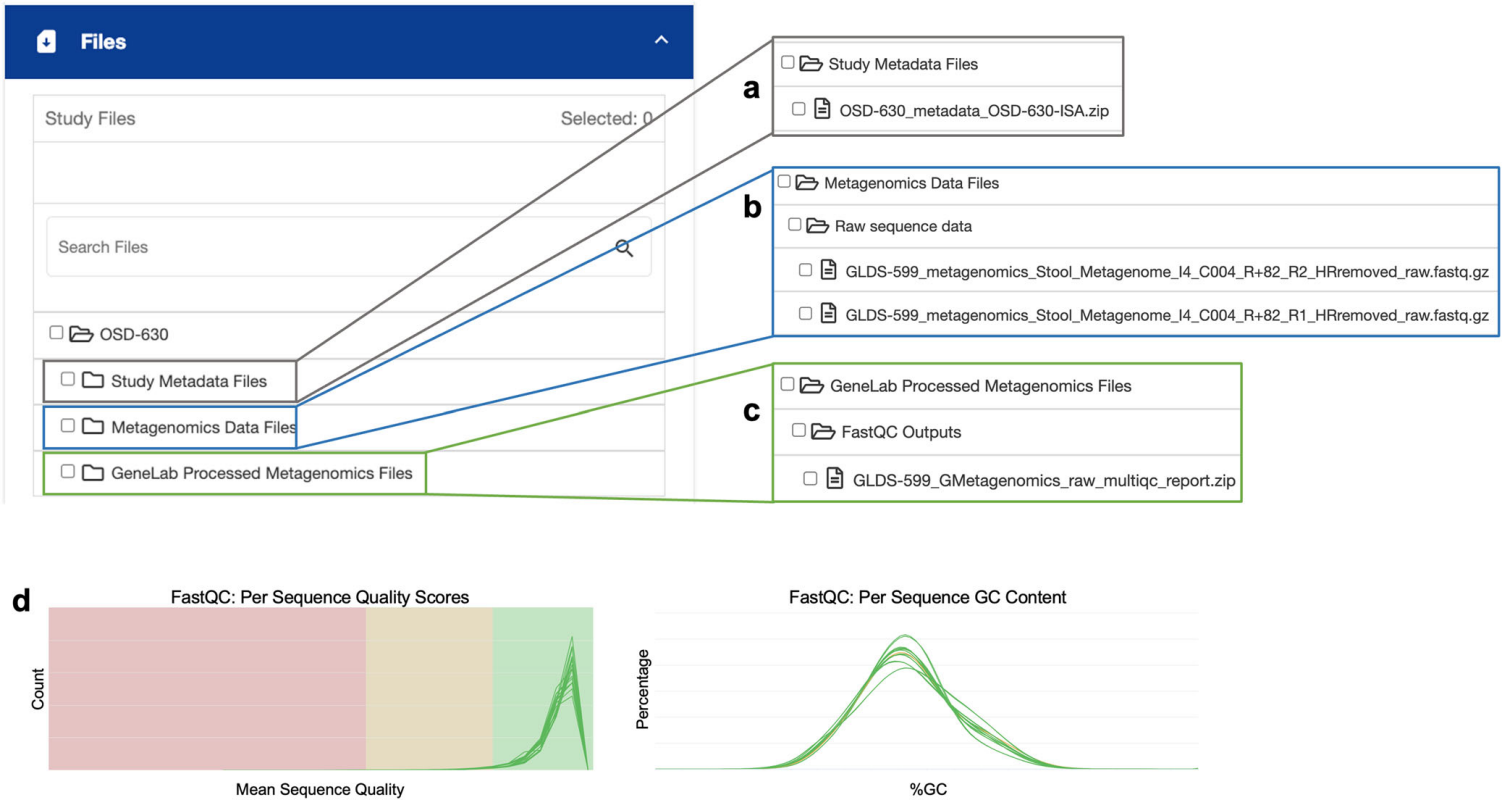
Data available for browse and download in OSDR for I4 study OSD-630. **a** Study metadata files include ISA-Tab files. **b** Investigator-deposited data files include the Metagenomics Data Files containing (human-read removed) raw FASTQ sequence data files and any investigator processed data files. **c** GeneLab Processed Metagenomics Files include a number of quality control outputs, including the FASTQC Outputs with a raw MultiQC report, and will contain GeneLab processed data files. **d** Examples of MultiQC data available for OSD-630.

Figure 3 shows the raw and processed data files available to browse and download through the OSDR study page for OSD-630. All OSDR datasets use the ISA-Tab (Investigation, Study, Assay) metadata model^6^ to capture experimental metadata for each study. Under the “Study Metadata Files” folder, users can download the *ISA.zip file for the study, which contains the ISA-Tab files that hold all study information displayed on OSDR, to enable further characterization of the dataset (Fig. 3a). Investigator-submitted data, both raw and processed, can be found under “Metagenomics Data Files”. This folder houses raw FASTQ sequences, which are essentially the sequence files post-removal of human-read data (see Fig. 3b for visual representation).

Furthermore, GeneLab, an integral component of the Open Science Data Repository (OSDR), has devised standardized omics data processing pipelines. These pipelines are instrumental in generating and disseminating processed data outputs for all omics datasets. They incorporate a rigorous quality control assessment process, including tools such as FastQC and MultiQC, particularly tailored for sequencing data analysis (refer to Fig. 3c, d for an illustrative depiction).

Moreover, it is noteworthy that the OSDR encompasses a diverse array of physiological and phenotypic data types, spanning cytokine panels, complete blood counts, imaging data, ultrasonography records, and more. These data types are subject to stringent data and metadata standards, complemented by tabular data transformation protocols. This meticulous approach is designed to uphold data quality and foster data reuse. Importantly, these physio-phenotypic standards have been developed and reviewed in collaboration with subject matter experts who specialize in the respective assays, forming an integral part of the OSDR Analysis Working Groups (AWG).

## Usage notes

These data, like any other datasets deposited into the OSDR repository, can be programmatically accessed through GLOpenAPI (https://visualization.genelab.nasa.gov/GLOpenAPI/). Here, we provide a quick example of running a differential expression analysis and pathway enrichment visualization in R (https://www.R-project.org/) using the unnormalized counts data associated with study OSD-569 (whole blood direct RNA-seq data).

However, please note that unlike most other datasets, access to human RNA-Seq data requires authentication with sufficient credentials, which may be made available upon request. I.e. while normally one would simply use the read.csv() function, for these data, we will define a get.csv() function that passes the authentication token in the header of the request; we assume the token is stored in a variable called “TOKEN”:


library(httr)get.csv = function (url, …) {auth = paste("Bearer", TOKEN) response = GET(url, add_headers(Authorization=auth)) return(read.csv(text=content(response), …))}


### Metadata

Since dataset OSD-569 is of interest, a query component “id=OSD-569” should be passed, and to retrieve all factor value metadata, a component “study.factor value” is added. Sample names and factors are columns in the metadata table. The header of the metadata table consists of two lines, and the first one may be skipped in many cases (see the GLOpenAPI manual for full explanation). The sample name is always the 3rd column (after “accession” and “assay name”), but, for illustrative purposes, here, we read everything as regular columns and assign row names manually in a later step. The request is sent to the GLOpenAPI “metadata” endpoint:


GLOPENAPI = "https://visualization.genelab.nasa.gov/GLOpenAPI/"query = URLencode("id=OSD-569&study.factor value")url = paste0(API, "metadata/?", query)metadata = get.csv(url, skip=1)cat("Metadata column names:\n")print(colnames(metadata))


Output:Metadata column names:

[1] "X.accession" "assay.name"

[3] "sample.name" "subject"

[5] "timestamp"

### Data

The same query component is included to retrieve data for OSD-569 (“id=OSD-569”). For differential expression analyses, data from files containing unnormalized RNA-Seq counts is requested (component “file.datatype=unnormalized counts”); the request is sent to the GLOpenAPI “data” endpoint.

As it is common for differential expression analyses, sample names are column names in the data table, and gene names are row names. Gene names always come first, so we pass “row.names=1” to the get.csv() function.

The header of the data table consists of three lines (accession, assay name, sample name), but as we are working with a single assay, we can skip the first two lines:


q = "id=OSD-569&file.datatype=unnormalized counts"url = paste0(GLOPENAPI, "data/?", URLencode(q))data = get.csv( url, check.names=F, skip=2, row.names=1)


### Differential expression and pathway enrichment analysis

At this point, the data is ready to be ingested into standard analysis tools, for example, DESeq2^7^and fgsea^8^ with the use of MSigDb^9^ pathways. For DESeq2, sample names in the metadata should be represented as row names; and if, for example, we want to classify all timestamps into “preflight” (anything before launch, i.e. timestamps starting with “L-“), “postflight” (immediately after landing, “R+1”) and “recovery” (all other timestamps starting with “R+“), we also add a “status” column:


rownames(metadata) = metadata$sample.namemetadata$status = sapply(metadata$timestamp, function (t) {


ifelse(t=="R+1", "postflight",

ifelse(startsWith(t, "R+"), "recovery",


 "preflight"))})


**Fig. 4:**
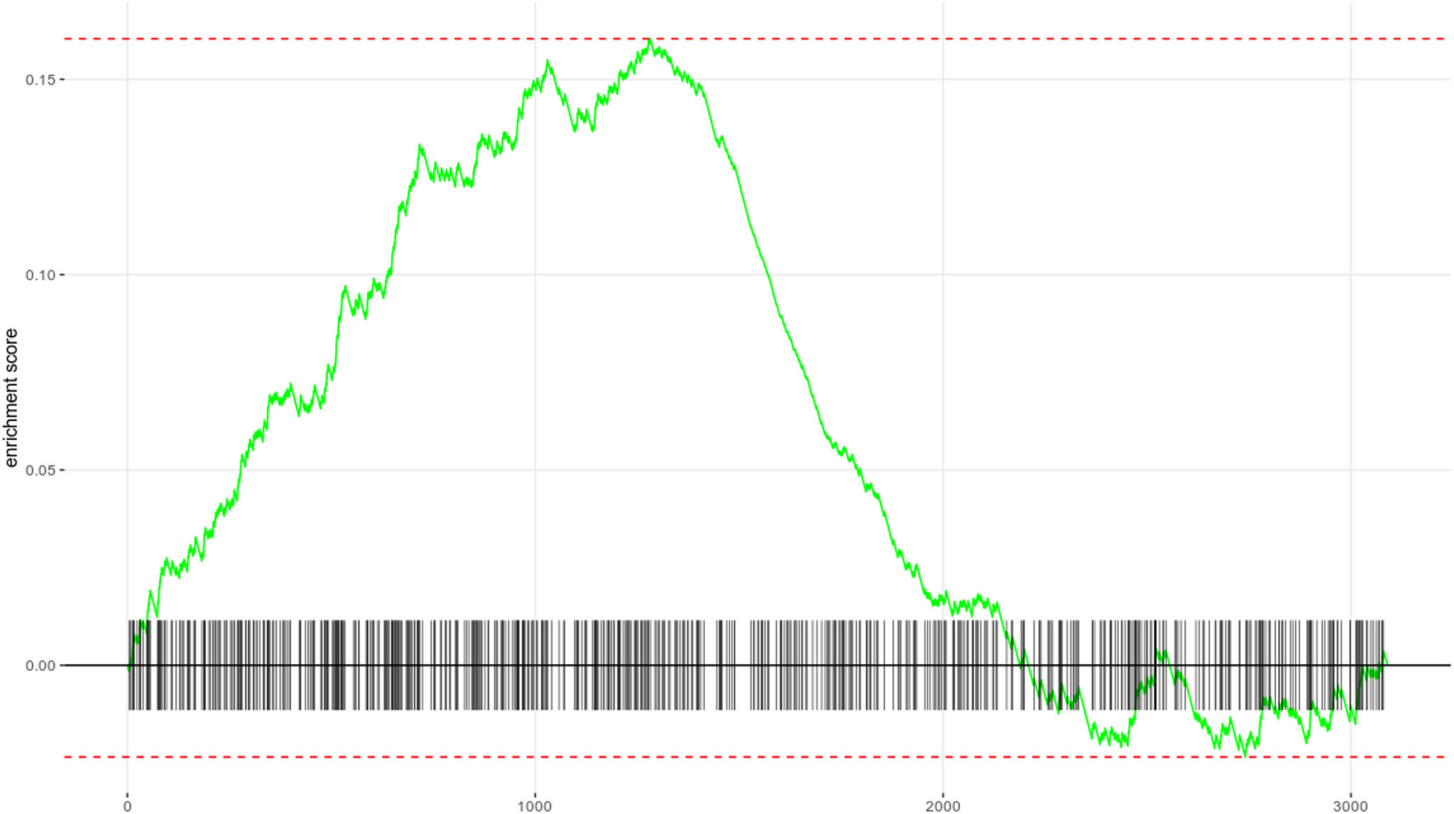
Enrichment analysis. Enrichment of the DIAZ_CHRONIC_MYELOGENOUS_LEUKEMIA_UP MSigDb pathway in the RNA-Seq data associated with dataset OSD-569 at the postflight timestamp compared to preflight conditions, analyzed and visualized from data retrieved via GLOpenAPI.


Finally, we run the analyses and visualize enrichment of a particular MSigDb pathway (DIAZ_CHRONIC_MYELOGENOUS_LEUKEMIA_UP) with result displayed in Fig. 4:

library(DESeq2)dds = DESeqDataSetFromMatrix( round(na.omit(data)), # DESeq2 requires integer values and absence of NaNs metadata, ~status+subject,)


lrt = DESeq(dds, test="LRT", reduced=~subject)


deg = results(lrt, contrast=c("status","postflight", "preflight"))library(msigdbr)


C2 = msigdbr(species="human", category="C2")

pathways = split(x=C2$ensembl_gene, f=C2$gs_name)


library(fgsea)deg$rank = deg$padj * sign(deg$log2FoldChange)ranks = with(na.omit(deg),setNames(rank, rownames(na.omit(deg))))plotEnrichment(pathways$DIAZ_CHRONIC_MYELOGENOUS_LEUKEMIA_UP, ranks)


## Technical validation

The establishment of standardized data formats, uniform units of measurement, ontological frameworks, and comprehensive metadata fields is imperative to ensure the seamless integration of new data types into a repository and to enhance the capacity for cross-study biological analyses. Further, the integration of medical assays with multi-omics data, including genomics, proteomics, metabolomics, and other data modalities, facilitates meta-analyses geared towards better understanding of the human response to the space environment. The correlation between omics data and clinically significant endpoints derived from medical data holds paramount significance in elucidating health implications. Nonetheless, the integration of disparate data types remains a persistent challenge in the biomedical research domain, exacerbated by inconsistencies across laboratories and studies, as well as inherent variability in individual responses. Moreover, space missions like I4, which encompass multiple data collection points (pre-flight, in-flight, post-flight, etc.), provide longitudinal datasets that enable the intricate yet potent exploration of space-induced responses over time.

Over the five years, the OSDR Analysis Working Groups (AWGs) were convened in order to ensure the reusability and standardization of data available within OSDR. These AWGs consist of diverse OSDR data stakeholders, including investigators, commercial entities, academic researchers, and students. Each AWG is dedicated to a distinct data category or domain of expertise, including animals, artificial intelligence, microbes, multi-omics, phenotypic data, and plants. Professionals within these AWGs contributed their expertise towards the formulation of standardized data processing pipelines and the development of assay metadata configurations tailored to each new data type. Consequently, OSDR was well-equipped to receive, curate, standardize, and process the wide spectrum of medical and omics data collected during the I4 mission.

Medical data serves as a crucial resource for gaining insights into biological responses. However, balancing the imperative to protect sensitive health information of astronauts while enabling data sharing for research necessitates the robust implementation of privacy and security protocols. OSDR has introduced an innovative capability, allowing users to access metadata associated with private medical datasets, albeit without direct access to the data itself. OSDR’s dedication to comprehensive and unambiguous metadata, coupled with the utilization of the GLOpenAPI, underscores its readiness for analysis and artificial intelligence applications. The data query method illustrated here marks an initial step toward realizing the goal of Precision Space Health^10,11^. In cases where users seek to employ private medical datasets such as those collected on I4 for research purposes, an application process is in place that mandates approval from an institutional review board (IRB).

### Supplementary information

Reporting Summary

## Data Availability

All data are available on the NASA Open Science Data Repository. OSD-569, OSD-570, OSD-571, OSD-572, OSD-573, OSD-574, OSD-575, OSD-630, OSD-656, OSD-687:

## Code availability

GeneLab processed data is generated using the pipelines that are publicly available on the GeneLab Data Processing GitHub page: https://github.com/nasa/GeneLab_Data_Processing.
